# Small and Long Non-Coding RNA Analysis for Human Trophoblast-Derived Extracellular Vesicles and Their Effect on the Transcriptome Profile of Human Neural Progenitor Cells

**DOI:** 10.3390/cells13221867

**Published:** 2024-11-11

**Authors:** Jessica A. Kinkade, Pallav Singh, Mohit Verma, Teka Khan, Toshihiko Ezashi, Nathan J. Bivens, R. Michael Roberts, Trupti Joshi, Cheryl S. Rosenfeld

**Affiliations:** 1 Biomedical Sciences, University of Missouri, Columbia, MO 65211, USA; kinkadeja@missouri.edu; 2 Christopher S Bond Life Sciences Center, University of Missouri, Columbia, MO 65211, USA; mvkpb@missouri.edu (M.V.); toshihiko.ezashi@missouri.edu (T.E.); 3 MU Institute of Data Science and Informatics, University of Missouri, Columbia, MO 65211, USA; pshy3@missouri.edu; 4 Division of Animal Sciences, University of Missouri, Columbia, MO 65211, USA; tekakhan@health.missouri.edu (T.K.); robertsrm@missouri.edu (R.M.R.); 5 Colorado Center for Reproductive Medicine, Lone Tree, CO 80124, USA; 6 Department of Genomics Technology Core Facility, University of Missouri, Columbia MO 65211, USA; bivensn@missouri.edu; 7 Department of Biochemistry, University of Missouri, Columbia, MO 65211, USA; 8 Department of Biomedical Informatics, Biostatistics and Medical Epidemiology (BBME), University of Missouri, Columbia, MO 65212, USA; 9 Department of Genetics Area Program, University of Missouri, Columbia, MO 65211, USA; 10 Department of Thompson Center for Autism and Neurobehavioral Disorders, University of Missouri, Columbia, MO 65211, USA

**Keywords:** brain, DOHaD, exosomes, in vitro models, lncRNA, miR, miRNA, placenta, RNAseq, transcripts

## Abstract

In mice, the fetal brain is dependent upon the placenta for factors that guide its early development. This linkage between the two organs has given rise to the term, the placenta–brain axis. A similar interrelationship between the two organs may exist in humans. We hypothesize that extracellular vesicles (EVs) released from placental trophoblast (TB) cells transport small RNA and other informational biomolecules from the placenta to the brain where their contents have pleiotropic effects. Here, EVs were isolated from the medium in which human trophoblasts (TBs) had been differentiated in vitro from induced pluripotent stem cells (iPSC) and from cultured iPSC themselves, and their small RNA content analyzed by bulk RNA-seq. EVs derived from human TB cells possess unique profiles of miRs, including *hsa-miR-0149-3p*, *hsa-302a-5p*, and many long non-coding RNAs (lncRNAs) relative to EVs isolated from parental iPSC. These miRs and their mRNA targets are enriched in neural tissue. Human neural progenitor cells (NPCs), generated from the same iPSC, were exposed to EVs from either TB or iPSC controls. Both sets of EVs were readily internalized. EVs from TB cells upregulate several transcripts in NPCs associated with forebrain formation and neurogenesis; those from control iPSC upregulated a transcriptional phenotype that resembled glial cells more closely than neurons. These results shed light on the possible workings of the placenta–brain axis. Understanding how the contents of small RNA within TB-derived EVs affect NPCs might yield new insights, possible biomarkers, and potential treatment strategies for neurobehavioral disorders that originate in utero, such as autism spectrum disorders (ASDs).

## 1. Introduction

While a transient organ, the placenta serves a vital role during gestation in that it acts as a bridge between the mother and fetus. However, the placenta not only provides physical contact and nutrient and gas exchange with the underlying uterine tissue, but pregnancy success itself is often dependent on placental trophoblasts producing factors that promote the survival of the ovarian corpus luteum, such that it continues to produce progesterone [1]. While the role of the placenta in guiding maternal physiology has been well studied, we are now just beginning to understand how it regulates aspects of fetal development and, in particular, the emerging brain [2,3,4].

The intricate connection between the placenta and the brain has been branded the placenta–brain axis [3,4]. The neurotransmitter serotonin (5-HT), for example, which accumulates in the parietal trophoblast giant cells (pTGCs) of the mouse placenta [5], has a pivotal role in shaping early brain formation. Dopamine is also found in the mouse placenta [5], although it has been understudied.

The mouse placenta also produces microRNAs (miRs) whose expression can be influenced by maternal bisphenol A (BPA) exposure [6]. The mRNA transcripts inferred to be affected by such miRs are, surprisingly, ones primarily expressed in neural tissues and involved in neurogenesis and differentiation pathways [6]. Other investigators have reported associations between placenta-derived miRs and the regulation of fetal neurodevelopment in both mice and humans [7,8,9]. A human cohort study showed correlations between the kinds of placental miRs/mRNA and intellectual/social impairments [8], providing compelling evidence that miRs serve as another link in the placenta–brain axis. The deletion of the mammalian-specific *miR379-410* cluster in the mouse placenta results in hyper-social behavior increased excitatory synaptic transmission, and the enhanced expression of ionotropic glutamate receptor complexes in the hippocampus [9]. The results suggest that this miR cluster expressed in placental tissue acts on the brain.

It is unclear how such small RNAs and monoamines elude fetal metabolism to achieve functional concentrations in the emerging brain. However, extracellular vesicles (EVs) are membrane-bound structures known to transport various kinds of RNA, proteins, and other informational molecules from their sites of production to various target sites, which is a likely possibility. EVs are sub-classified into microvesicles, exosomes, and apoptotic bodies [10]. Transmission electron microscopy and omics approaches can be used to identify and analyze such structures and have demonstrated distinct cell-of-origin signature patterns.

Several studies have examined how placenta-derived EVs or small RNAs contained within EVs influence the mother and/or change the disease state (preeclampsia and gestational diabetes) [11,12,13,14,15,16,17,18,19,20,21,22,23,24,25,26,27]. It is clear that miRs within placental EVs fluctuate throughout pregnancy, and the expression patterns of some of these miRs are linked with eventual birth weight [28]. The miRs and protein content of placenta-derived EVs may regulate inflammation and trophoblast invasion [29] and stimulate acute and chronic inflammation, leading to fetal brain damage [30]. EVs from the human placenta have been previously shown to affect the transcriptome and other properties of skin fibroblasts [31]. We recently characterized the protein, miRs, serotonin, and catecholamines within EVs derived from mouse trophoblast stem cells (TSCs) and the TSCs that differentiate into parietal trophoblast giant cells (pTGC) [32]. We have also shown that the exposure of mouse neural progenitor cells to EVs derived from either TSC or pTGC impacts the transcriptome profile of these cells.

As most studies to date have focused on how human TB-derived EVs and miRs influence maternal physiology, we sought to characterize how the small RNA content of EVs derived from human TB cells differentiated from induced pluripotent stem cells. Secondly, we examined whether human NPCs derived from the same iPSC internalize such EVs and whether this treatment affects their transcriptome profile. By characterizing the miRNA content of human TB-derived EVs and establishing potential linkages to human NPC transcriptome changes, our goal has been to provide a better understanding of how EVs function as go-betweens in the placenta/brain dialog.

## 2. Materials and Methods

### 2.1. Cell Lines

Experiments were performed on cell lines generated in the Roberts laboratory. These were derived from primary cultures of outgrowths from the minced umbilical cord tissue of infants after normal pregnancies [33] and subsequently reprogrammed into iPSC with episomal plasmids [34]. The iPSC line used for the described studies, “MRuc5i” was from a female infant born after a normal pregnancy at 39.5 weeks [34] and could be readily converted to a range of TB cell types by using the widely used BAP differentiation protocol (BMP4/A83-01/PD/PD173074) [35,36,37,38,39]. At day 6 of differentiation, the colonies, which could reach a diameter of 0.5 cm or greater, comprise a mixture of mononucleated cytoTB, extravillousTB, and synctioTB (STB) [40]. Thus, EVs collected from these cultures represent a mixture of TB cell types, all of which are represented in the early placenta.

We derived neural progenitor cells (NPCs) [41] from the same iPSC MRuc5i line used for TB differentiation. These cells ultimately functioned as the recipients in EV uptake experiments. The conversion of iPSC to NPC was conducted through a monolayer culture by using the STEMdiff^TM^ Neural Induction Medium (StemCell Technologies, Vancouver, Canada) [39]. Clonally propagated cells were validated for neural differentiation as described for the cell line CTR 2 in Mullegama et al.’s study [41], working with iPSC lines that had been generated from skin fibroblasts. The NPC line used here (MRuc5NPC) was expanded on the STEMdiff^TM^ Neural Progenitor Medium and had the potential to differentiate to a range of neuronal cell types, although, in the present project, we focused on the undifferentiated NPCs.

### 2.2. Isolation and Fluorescent Labeling of EVs Derived from Human iPSCs and TBs

The EVs were harvested from progenitor iPSCs (controls) and iPSCs that had differentiated into TBs by the BAP procedure described previously. To characterize the EVs from a mixture of human TBs, the spent medium was collected from the medium of BAP-differentiated colonies between days 5 and 6 of the BAP treatment, as well as from undifferentiated controls. The supernatant solutions were first subjected to centrifugation (3000× *g* for 15 min) to remove large particles. EVs were isolated from the filtrates using an exosome isolation kit (ExoQuick-TC Ultra EV Isolation Kit for Tissue Culture Medium; ThermoFisher Scientific, St. Louis, MO, USA) per the manufacturer’s protocol. Partially purified EV pellets were resuspended in a mixture of Buffer A and B in the kit and used immediately. Transmission electron microscopy (Jeol JEM-1400; JEOL USA Inc., Peabody, MA, USA) was performed on copper formvar/carbon grids after 2% paraformaldehyde and uranyl acetate counterstaining confirmed EV morphology, relative purity, and the size of particles. We used NanoSight analyses to determine the concentration and average size of the EVs.

### 2.3. Small RNA Isolation from EVs Derived from Human iPSCs and TBs

miRs and mRNAs were isolated with the EVeryRNA™ EV RNA Purification System with ExoQuick^®^ EV Isolation (System Biosciences, Palo Alto, CA, USA). This kit should isolate all RNAs, including long non-coding RNAs (lncRNAs). The RNA concentration was analyzed on the 5200 Fragment Analyzer (Agilent Technologies, Santa Clara, CA, USA) at the MU Genomics Technology Core Facility.

### 2.4. Small RNA Library Preparation for EVs from Human iPSCs and TBs

The Genomics Technology Core Facility at the University of Missouri performed the small RNA sequencing analysis. Libraries were constructed using the manufacturer’s protocol with reagents supplied in the SMARTer small RNA library preparation kit (cat # 635029, Takara Bio USA, Inc., San Jose CA, USA). Total RNA was first polyadenylated to provide a priming sequence for an oligo(dT) primer. First-strand synthesis was performed with PrimeScript™ Reverse Transcriptase primed by the 3′ smRNA dT Primer. Non-templated nucleotides were added at the 3′ end of the first-strand cDNA molecule to be bound by the SMART smRNA Oligo. The PrimeScript Reverse Transcriptase used the SMART smRNA Oligo as a template for template switching and extension. Full-length Illumina adapters containing indexes were then added by PCR amplification. Amplified small RNA libraries were purified with NucleoSpin Gel and a PCR clean-up kit, and libraries were further enriched by using AxyPrep MAG purification beads (Axygen, Inc. Union City, CA, USA), which performed a double-sided size selection (bead to sample ratio: 0.8×:2.0×) to remove adapter dimers and to recover the desired insert size of 18–45 bases. The DNA fragments (libraries) were recovered by elusion from the bead-bound fraction in 22 µL of a 10 mM Tris Buffer, pH 8.5. The amount of DNA in each library was quantified with the Qubit HS DNA kit (Life Technologies, Carlsbad, CA, USA)), and the fragment size was analyzed by an Agilent 5200 Fragment Analyzer. The amount of DNA in each library was quantified with the Qubit HS DNA kit (Life Technologies), Libraries were then pooled and diluted according to the standard sequencing protocol for sequencing on a NovaSeq 6000 (Illumina, San Diego, CA, USA).

### 2.5. Small RNAseq Data Processing and Analysis

The initial quality screening was conducted with the Cutadapt program (v 4.6) [42] to remove the first three nucleotides, poly-A adapters, Illumina adapters, ambiguous nucleotides (N’s), paired sequence reads shorter than 20 bp, and reads with a Phred score less than 20. After trimming, the paired-end reads were concatenated for miRNA analysis with miRge3 [43], which was enhanced with miRNA Error Correction to generate raw read counts. Differential expression analysis was performed by means of DESeq2 [44] with a *p*-value threshold of 0.05 and an absolute fold change of 2. The miRNA TissueAtlas2 program updated 23 July 2022 [45] was then used to examine the tissue and organ enrichment for individual miRs that were differentially expressed in EVs from TB cells relative to those from iPSC. This program, however, does not include the placenta.

The TissueEnrich program (Version 1.26.0) [46] was used to determine which human organs and tissues have an abundance of mRNA transcripts that might be recognized by differentially expressed miRs. ClueGO (Version 2.5.10) [47], which is a Cytoscape (Version 3.10.3) plug-in, was used to determine gene ontology and pathways that are predicted to be affected by mRNAs targeted by differentially expressed miRs.

The human reference genome (GRCh38) from GENCODE [48] and GFF3 files from the RNACentral [49] database were used to extract lncRNAs from the sequencing data. Bowtie [50] was used for the read alignment and feature counts [51] and for extracting raw read counts. DESeq2 [44] identified differentially expressed lncRNAs with an adjusted p-value threshold of less than 0.05 and a log2 fold change of ≥ 2, yielding 6794 lncRNAs. We mapped the RNACentral Identifiers to lncPedia [52] and lncBook [53].

To understand the interactions between miR and lncRNA changes in TB-derived EVs and transcriptomic changes in NPC t (methods detailed below), we used NcPath [54], which provides KEGG [55] pathway associations. We created an interaction network by using the igraph package [56] in R [57], which was refined and annotated with RCy3 [58] and loaded into Cytoscape [59] for enhanced visualization. This approach provided insights into the complex regulatory networks involving miRs, lncRNAs, and their target genes. Finally, we employed the TissueEnrich program [46] to determine which tissues have the enrichment of the 185 intersecting transcripts differentially expressed in their NPCs when treated with EVs and miRs/lncRNAs differentially expressed in TB EVs.

### 2.6. Internalization of EVs from Human TB Cells by Fetal Neural Stem Cells

Invitrogen BODIPY™ TR Ceramide (ThermoFisher Scientific, St. Louis, MO, USA) was used to pre-label exosomes for uptake studies. These experiments were performed on four separate occasions over a period of six weeks. For each isolated EV sample, 1 µL of dye was added for every 100 µL isolated EVs for a final dye concentration of 10 µM. EVs were incubated with the dye in the dark at 37 °C for 20 min. Excess dye was removed from the tagged EVs using the same spin column employed originally.

MRuc5NPCs were cultured in 2 mL of a medium in TPP 6-well bottom polystyrene plates (TPP, Trasadingen, Switzerland) and exposed to labeled EVs when they were ~50% confluent (approx. 24 h after passage). Unexposed cells served as negative controls. Pilot studies with EVs from H1 embryonic stem cells (ESC, WiCell: WA01) differentiated for mixed TB populations with BAP treatment, which were run to demonstrate that the small amounts of EVs employed caused no overt toxicity and did not affect the proliferation rate. A time course revealed that an incubation period of one hour with EVs was sufficient to provide adequate uptake of the label for imaging, which is similar to that previously found for mouse NPCs exposed to EVs derived from mouse TSCs and those differentiated into pTGC [32]. In the experiments with MRuc5NPC, the presence of labeled EVs associated with the cells was assessed by fluorescent imaging at 0, 20, 40. and 60 min after exposure with a Leica TCS SP8 confocal system (Leica Biosystems, Deer Park, IL, USA). A three-dimensional (3D) animation of the cells was acquired with the LAS X software program (Version 5.2.2) (Leica Microsystems).

### 2.7. RNA Isolation from Human NPCs

In parallel experiments, separate groups of MRuc5NPCs were exposed to unlabeled EVs from BAP-treated iPSCs differentiated into TBs and progenitor iPSCs for 24 h, and RNA was isolated with the Qiagen AllPrep DNA/RNA/miRs Universal Kit (catalog #80224; Qiagen, Germantown, MD, USA). This time period was chosen based on our previous work that showed mouse NPCs exposed to EVs from mouse TSCs or pTGCs, which demonstrated significant gene expression changes after 24 h of treatment [32]. The quantity and quality of the RNA were determined with a Nanodrop ND1000 spectrophotometer (Nanodrop Products, Wilmington, DE, USA). The results were further confirmed by analyzing the RNA on the Fragment Analyzer (Advanced Analytical Technologies, Ankeny, IA, USA). Only those samples that had an RNA integrity number (RIN) score > 8.0 were selected for RNA sequencing (RNAseq).

### 2.8. Illumina RNA Library Preparation and Sequencing

Libraries were constructed by the manufacturer’s protocol with reagents supplied in Illumina’s mRNA Stranded Library Preparation Kit and sequenced at the University of Missouri Genomics Technology Core. In brief, poly-A, containing mRNA was purified from total RNA using poly-T oligo beads, mRNA fragments, and double-stranded cDNAs generated from cleaved RNA using random hexamers as the primers. cDNAs then underwent end-repair and adapter ligation followed by PCR amplification to selectively amplify the anchor-ligated DNA fragments and to add unique dual indexes.

The quantity and purity of the final library were determined on an Agilent Fragment Analyzer quantified with the Qubit fluorometer by means of the Qubit dsDNA HS Assay kit (Thermo Fisher Scientific) and diluted according to Illumina’s standard sequencing protocol. Libraries were pooled and run on an Illumina NovaSeq 6000 sequencer with a paired-end 100 bp read format on an S4 flow cell to generate ~100 million paired reads per sample.

The raw RNASeq reads’ quality check was performed using FastQC (Version 0.11.9) [60] and aggregated through the MultiQC tool (Version 1.11) [61]. The reads were later trimmed with Trim Galore (Version 0.6.7), which is a wrapper for Cutadapt for removing Illumina adapters, ambiguous nucleotides (N’s), any sequence reads with a total read length <20 bp, and a quality Phred score of 20, which is calculated using −10 × log_10_(Probability of Incorrect Base Call) to ensure a base call accuracy of 99% for the sequencing. The trimmed reads were further aligned to the reference human genome (GRCh38.108) with HISAT2 (Version 2.2.1) [62] to achieve a high overall alignment (~93.5%). The aligned files were converted to sorted bam files by means of Samtools (Version 1.14) [63] and further processed with Cufflinks (Version 2.2.1) [64] to generate gene expression abundance levels.

### 2.9. Differential Gene Expression Analysis (DGEA): Cufflinks

Differential gene expression analysis (DGEA) was carried out by the Cuffdiff method [64], available through the Cufflinks package (Version 2.2.1). Genes were considered upregulated or downregulated in human NPCs treated with EVs from human TBs vs. vehicle controls; those treated with EVs from human iPSCs vs. vehicle controls; and those treated with EVs from TB cells vs. iPSCs if they had an absolute fold change ≥ 1.5 and *q*-value ≤ 0.05. Volcano and PCA plots were drawn using the Enhanced Volcano package (Version 1.16.0) [65] and the Princomp method available through R (Version 4.2.2) [57], respectively.

### 2.10. Protein–Protein Interactions

Protein–protein interactions (PPIs) for proteins encoded by DEGs for the three comparisons were determined by the STRING Database [66]. The PPI files generated with STRING were acquired and loaded in Cytoscape [59] to examine the top 10 hub genes with the Cytohubba app (Version 0.1) [67]. Within this program, hub genes were identified with the Maximal Clique Centrality (MCC) method as default parameters [67].

### 2.11. Gene Functional Enrichment, Brain-Specific Gene Enrichment, and Network Analyses

For functional enrichment analysis, DEGs were imported into the WEB-based GEne SeT AnaLysis Toolkit (WebGestalt) 2019 version and the gene ontology molecular function (GO MF) and gene ontology biological processes were searched for (GO BP) [68]. Brain-specific gene enrichment analysis for differentially expressed genes was determined by the GTEx Portal (API V2) [69]. This was performed for the top 50 differentially expressed genes in each of the comparisons by searching all brain regions in this database.

## 3. Results

### 3.1. Small RNAseq Analyses of Small RNAs Within EVs Derived from Human TBs or iPSCs: General Features

**Figure 1:**
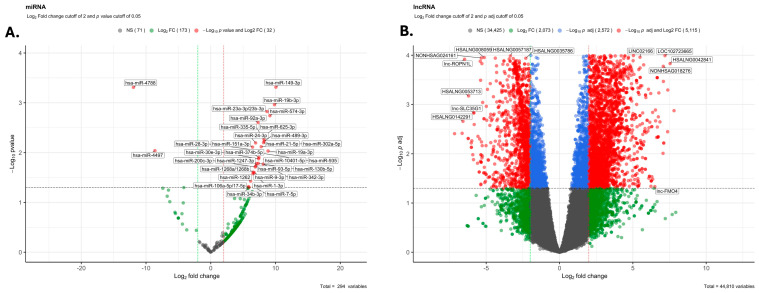
Volcano plots for miRs and lncRNAs within EVs from TB vs. iPSC. Gray dots are miRs or lncRNAs that are not differentially expressed. (**A**) Volcano plot depicting the differential expression of microRNAs (miRs) within extracellular vesicles (EVs) from TB versus iPSC groups. Gray dots represent miRs that are not differentially expressed. Green dots indicate miRs with a log2 fold change difference between TB and iPSC groups, while red dots highlight miRs with both a significant log10 *p*-value and log2 fold change difference. (**B**) Volcano plot for long non-coding RNAs (lncRNAs) within EVs from TB versus iPSC groups. Gray dots denote non-differentially expressed lncRNAs. Green dots show lncRNAs with a log2 fold change between the two groups, and red dots represent lncRNAs with a significant log10 *p*-adjusted (*Q*)-value and log2 fold change difference.

The small RNAseq reads for each sample and the distinct types of small RNAs are provided in Supplementary Table S1. As shown, the average number of trimmed reads for iPSCs and TBs were 24,722,027 and 22,195,525, respectively. The average number of miRs for iPSC and TB were 136,277 and 122,500, respectively. For lncRNAs, the values were 3,740,063 and 1,498,884, respectively. The values for additional small RNA reads are provided in Supplementary Table S1. Supplementary Figure S1A illustrates the breakdown for each sample and type of small RNA. Based on all miRs and lncRNAs, there is no clear clustering between EVs derived from iPSCs vs. those derived from TB cells (PERMANOVA value = 0.1 for miRs and lncRNAs), as shown in the PCA plot (Supplementary Figure S1B) and heat maps (Supplementary Figure S2A–C). However, the variability across samples is minimal. The use of z-scores for visualizing the Fragments Per Kilobase per Million mapped fragments (FPKM) data, while effective for highlighting differences, can exaggerate minor fluctuations, especially when variability is low, as discussed in [44,70]. Z-score transformation standardizes the data into units of standard deviation from the mean, which can amplify small differences and make modest changes appear more significant in the visualizations. However, differential gene expression analysis is based on *q*-values < 0.05, ensuring a false discovery rate of less than 5%, which maintains the statistical robustness of the findings. The Volcano plots show that certain miRs and several lncRNAs differed between these two groups (Figure 1).

### 3.2. Small RNA Differences Between EVs Derived from Human TB Vs. iPSCs

**Figure 2:**
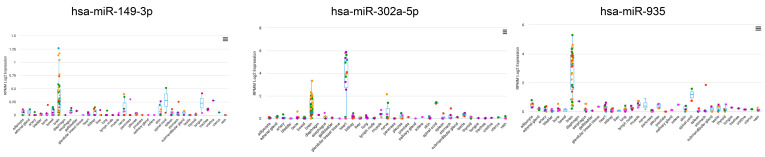
The analysis of individual miRs with the miRsTissueAtlas2 program [45]. The diagram shows that hsa-miR0149-3p is predominantly expressed in the brain, hsa-miR-302a-5p is abundantly expressed in the heart, followed by the brain and nerve tissues, and hsa-miR-935 is almost exclusively expressed in the brain.

The miRge3 program (Version 0.1.3) [43], followed by DESeq2 [44], was used to analyze miR differences between EVs isolated from TB Vs. iPSC. Based on a *p*-value threshold of 0.05 and a log2 fold change of 2, 32 differentially expressed miRs were identified, of which 5 displayed a *p*-adjusted value (FDR) of ˂0.05 and an absolute fold change greater than 2 (Supplementary File S1). The top 15 of these miRs are listed in Table 1. Only two miRs (*hsa-miR-4788* and *hsa-miR-*4497) were downregulated in TB-derived EVs relative to iPSC-derived EVs. The most abundantly expressed miRs in TB EVs vs. iPSC EVs listed in Table 1 were analyzed using the miRsTissueAtlas2 program [45], which, unfortunately, did not include the placenta in its database. This evaluation revealed that, while most of the miRs exhibited a wide tissue distribution, *hsa-miR0149-3p* and *hsa-miR-935* were predominantly expressed in the brain, and *hsa-miR-302a-5p* in the heart, brain, and neuronal tissues (Figure 2). Analysis with the program miRPathDB (Version 2.0) also indicated that the primary GO biological processes affected by *hsa-miR0149-3p* are nervous system development, anatomical structure, morphogenesis, neurogenesis, the generation of neurons, and positive regulation of gene expression (Supplementary File S2).

To analyze lncRNAs and other small RNA differences, we utilized the human reference genome (GRCh38) from GENCODE [4] and GFF3 files from the RNACentral [5] database. DESeq2 was then used to identify differentially expressed lncRNAs in EVs from TB cells relative to iPSCs (with an adjusted *p*-value threshold of ˂0.05 and a log2 fold change of 2). This analysis revealed that 6794 lncRNAs were differentially expressed, with about equal numbers upregulated and downregulated (Supplemental File S3), as illustrated in the volcano plot (Figure 1). Analysis of the chromosomal distribution for the differentially expressed miRs and lncRNAs revealed that most of the differentially expressed miRs were widely distributed across the genome (Supplementary Figure S3).

### 3.3. Target mRNA for miRs Within EVs Derived from Human TsB Vs. iPSCs

**Figure 3:**
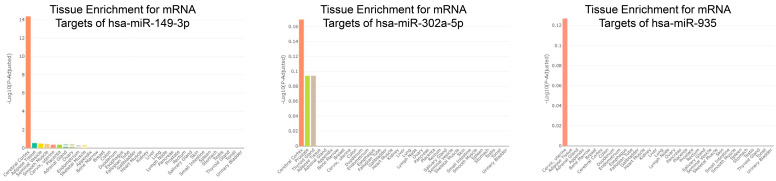
TissueEnrich program [46] analysis to determine which human organs and tissues have an abundance of transcripts that might be recognized by differentially expressed miRs shown in Figure 2. The primary mRNA targets for hsa-miR-0149-3p are enriched almost exclusively in the cerebral cortex. The mRNA targets for hsa-302a-5p are enriched in the cerebral cortex, followed by the prostate and thyroid gland. Primary mRNA targets for hsa-miR-395 are surprisingly abundant in the cervix and uterus.

Since miRs can regulate gene expression by pairing with particular mRNAs, usually in their 3^/^-termini, the https://mirdb.org (accessed on 20 October 2024) [71,72] was then used to predict the target transcripts of *hsa-miR0149-3p*, *hsa-miR-302a-5p*, and *hsa-miR-935* (Supplementary File S4). The TissueEnrich program (Version 1.26.0) [46] indicated that human organs and tissues have an abundance of transcripts that might be recognized by these miRs in EVs. The primary mRNA targets for hsa-miR-0149-3p are enriched almost exclusively in the cerebral cortex (Figure 3), while those for hsa-302a-5p are enriched not just in the cerebral cortex but also in the prostate and thyroid gland (Figure 3). Primary mRNA targets for *hsa-miR-395*, by contrast, are abundant in the cervix and uterus (Figure 3), suggesting that the primary targets might be in tissues of the maternal genital tract, including the endometrium. Other upregulated miRs in EVs from TB EVs include *hsa-miR-19b-3p* (potentially targeting transcript enriched in the cerebral cortex and skeletal muscle), *hsa-miR-23a-3p* (cerebral cortex, endometrium, and smooth muscle), and *hsa-miR-92a-3p* (cerebral cortex) (Supplementary Figure S4). The consideration of all mRNA targets for the differentially expressed miRs by the ClueGO program (Version 2.5.10) [47] reveals that they are associated with the following pathways: the positive regulation of viral transcription, spongiotrophoblast differentiation, histone H2A T120 phosphorylation, mitochondrial DNA repair and migration, DNA replication and ligation, and T helper cell responses (Supplementary Figure S5, Supplementary File S5).

### 3.4. Internalization of Human TBs and iPSC-Derived EVs by Human NPCs

**Figure 4:**
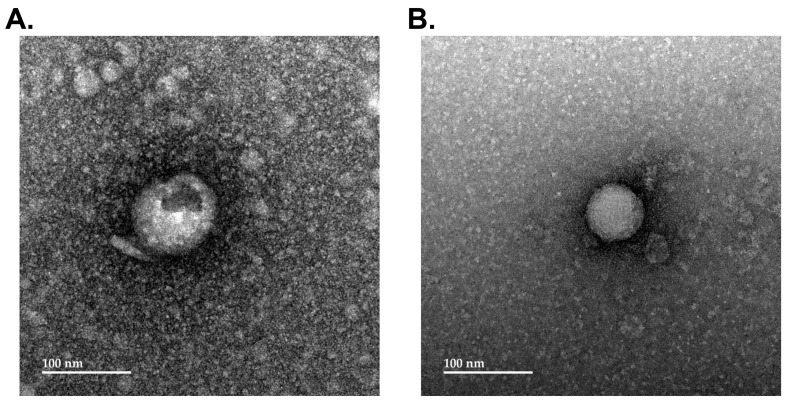
Extracellular vesicles (EVs) derived from human trophoblasts (TBs) and iPSCs and their internalization by human neural progenitor cells (NPCs). (**A**) A transmission electron microscopy (TEM) image of EVs derived from human TB cells. (**B**) A TEM image of EVs derived from human iPSCs.

As illustrated in Figure 4A, EVs from human TBs, counterstained with uranyl acetate, were membrane-bound and approximately 75–100 nm in diameter. The small punctate material around the EVs represents background staining with the uranyl acetate. As determined by confocal microscopy, the isolation of fluorescently labeled EVs yielded approximately 4.63 ± 0.84 EVs per 100 × high power fields for TB cells and 1.71 ± 0.42 EVs per 100 × high power fields for iPSC. Based on this information, human NPCs in the monolayer culture were exposed to 500–1500 EVs/µL of the cell culture media. The NPCs were visible as spindle-shaped cells characterized by cell projections labeled with Phalloidin 488. In experiments with MRuc5NPC, the presence of labeled EVs associated with the cells was assessed by fluorescent imaging at 0, 20, 40, and 60 min after exposure.

**Figure 5:**
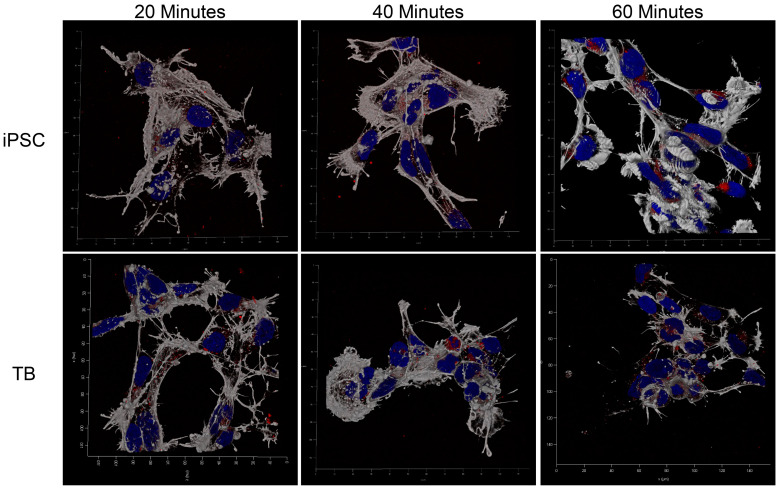
The internalization of EVs from TBs and iPSCs in human NPCs. (A) Fluorescence image of the internalization of EVs from human iPSCs. Red punctate material represents fluorescently tagged EVs (white arrows); the nuclei of NPCs are stained with DAPI (blue); and NPC fibers are labeled in green. (B) Fluorescence image of the internalization of EVs from human TB cells. Red punctate material represents fluorescently tagged EVs (white arrows); the nuclei of NPCs are stained with DAPI (blue); and NPC fibers are labeled in green.

Figure 5 and Supplementary Videos S1 and S2 show example images of the internalization of EVs by TBs and iPSCs after 40 min of exposure. Labeled EVs from both the parental iPSC (MRuc5i) and the same line converted to TBs became associated with the surface of the NPCs as early as 20 min after beginning the exposure. Over time, the label became largely concentrated in the perinuclear region of the cytoplasm.

### 3.5. General Features of Transcriptomic Data from Human NPCs Exposed to EVs from Human TB or Ipsc Vs. Non-Exposed NPCs

To provide the RNAseq data, RNA was isolated from the NPCs 24 h after they had been exposed to EVs. Four replicate samples from each treatment group were collected in separate experiments. The average number of total paired-end reads for all 12 samples of RNA collected was 34,941,582. There was an average of 93.5% alignment to the human genome, providing an average of 32,726,646 mapped paired-end reads (Supplementary Table S2). Based on our previous transcriptomic studies, this number of reads is more than sufficient to analyze eukaryotic transcriptome profiles [73,74,75,76,77,78,79].

**Figure 6:**
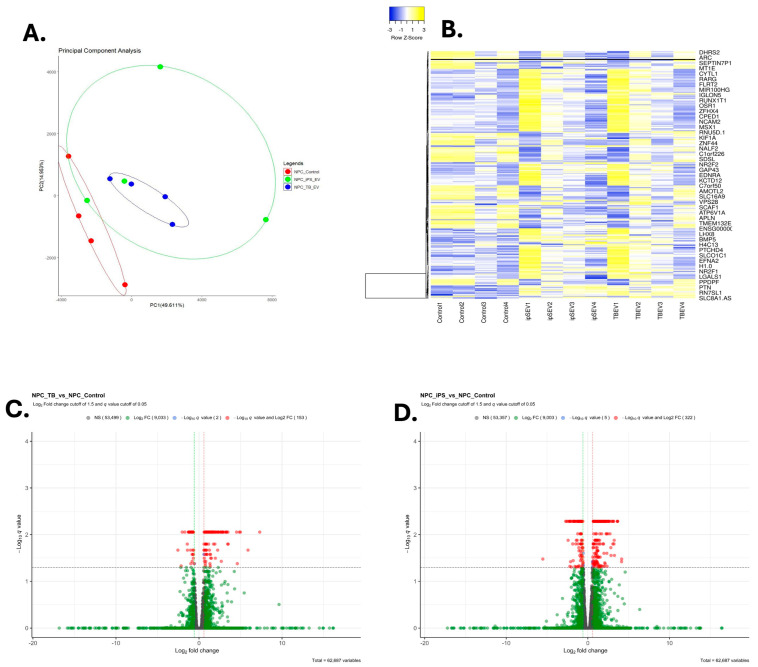
Transcriptome results of NPCs treated with TB EVs, iPSC EVs, and control NPCs. (**A**) A 2D PCA plot of NPCs treated with TB EVs (blue circles), iPSC EVs (green circles), and control NPCs (red circles). Clear separation is evident between control NPCs and those treated with TB EVs or iPSC EVs. (**B**) Heatmap analysis of NPCs treated with TB EVs, iPSC EVs, and control NPCs. The control NPC formed one cluster, whereas those treated with TB EVs and iPSC EVs showed some overlap between samples. (**C**) The volcano plot analysis of control NPCs vs. TB EVs treated with NPCs demonstrates several genes that show an increase of a more than 1.5-fold change (FC, shown in green), those few genes that have a −Log_10_ *Q*-value (equivalent to *q* value ≤ 0.05, shown in light blue), and those that qualified both a −Log_10_ *Q*-value and log_2_ FC (shown in red). (**D**) The volcano plot analysis of control NPCs vs. iPSC EVs treated with NPCs demonstrates several genes that show an increase of more than 1.5-fold change (FC, shown in green), those few genes that have a −Log_10_ *Q*-value (shown in light blue), and those that have qualified both a −Log_10_ *Q*-value and log_2_ FC (shown in red). Four independent replicates were assessed for each of the groups.

The 2D PCA plot revealed a separation of the transcriptomes of NPC controls and NPCs treated with EVs from TBs, whereas the transcriptomes obtained from the cultures of NPCs exposed to the isolates of EVs from iPSC (MRuc5i) are scattered across the diagram, indicating more inter-sample variability (Figure 6). The heat map analyses are consistent with these results and separated the sequence data into two major clusters (Figure 6), of which the first contained all four experimental replicates from controls not exposed to EVs, and the second contained the four replicates exposed to EVs from TBs. However, each of the two clusters also contained two RNA data sets derived from the NPCs exposed to EVs from the iPSC (Figure 6). The explanation for this variance among the iPSC samples is unclear. The same limitations in relation to the use of z-scores for visualizing FPKM data exist, as discussed previously [44,70].

### 3.6. Differential Gene Expression Profiles for Human NPCs Exposed to EVs from Human TBs or iPSCs Vs. Non-Exposed NPCs

All the genes differentially expressed across treatment groups based on a fold change ≥ 1.5 and q value (FDR) ≤ 0.05 are listed in Supplementary File S6. However, no significant differences were observed between NPCs treated with EVs from TB and those treated with EVs from control iPSC. There were 115 differentially regulated genes common to treatments with both types of EV, of which 102 were uniquely associated with treatment with EVs from iPSCs and 38 with EVs from TBs (Supplementary Figure S6).

Of the 153 transcripts differentially expressed between MRuc5iNPC iPSC controls (no EVs) and the same cells exposed to TB EVs for 24 h, the top 20 transcripts upregulated were ones for small cytoplasmic RNAs (*RNY1*, *-3*, *-4*; *RN7SL1*), a small nuclear RNA (*RNU5D*), two nuclear non-coding RNAs (*ENSG00000270103*, *ENSG00000289413*), two long intergenic non-coding RNAs (*LINC01102*, *LINC01579*), a small nucleolar RNA (*SNORD13*), and an antisense transcript for the sodium/calcium exchanger gene *SLC8A1-AS1*. Of these genes, *RNU5D* and *SLC8A1-AS1* have been linked to neuronal function or development. The remaining upregulated genes are among the top 20, including ones encoding three homeobox transcription factors (distal-less homeobox 1, *DLX1* and distal-less homeobox 2, *DLX2*, both of which are important in the survival of inhibitory neurons in the forebrain; and BARX homeobox-1, *BARX1*, best known for its role in craniofacial development). In addition, there was an upregulation of *SLCO1C2*, *DUSP9*, *IFI27L2*, and *SPOCK3*. Of these four, only the latter has been implicated in neurodevelopment.

The top 20 down-regulated genes for this comparison (*MGAT4B*, *PAK6*, *SLC22A18*, *MT2A*, *KIF1A*, *SH3TC1*, *XKR7*, *PCLO*, *VGF*, *EGR1*, *NGFR*, *ANKRD33B*, *ELFN1*, *PRR36*, *PLCB1*, *SDSL*, *APLN*, *C1orf226*, *SLC7A5*, and *CHST3*) are all ones implicated in one or more aspects of neuronal development. Three of the most recognizable (VGF, EGR1, and NGFR) encoded proteins with well-established roles in regulating neuronal cell growth.

Based on the same criteria (a fold change ≥ 1.5 and *q* value ≤ 0.05), 217 transcripts were differentially expressed between the control, i.e., untreated MRuc5NPC cells versus those treated with iPSC EVs. Of the top 20 upregulated genes, 7 (*DLX1, DLX2, BARX, DUSP9, SPOCK3, SLCO1C2,* and *LINC01102* were also upregulated in response to TB ERVs. Additionally, there was an upregulation of four long non-coding transcripts (*ENSG00000261786, ENSG00000265174, ENSG00000289413; LINC01579).* Six genes (*RORB, EDN3, CDKN1C, HMCN1, EMX2, SLC38A11)* have all been associated with certain aspects of neuronal development, while two (*SYNPO2* and *TUBGCP5*) are associated with cytoskeleton organization. Finally, *COL8A2* plays a role in the organization of the corneal basement membrane.

The top 20 downregulated genes again included *EGR1*, *VGF*, and apelin (*APLN*), which were also downregulated in response to EVs from TB, and an assortment of metallothionein (*MT1E*, *MT2A*, and *MT1F*) transcripts. Additional downregulated genes linked in some manner to neuronal development were *GMFG*, *TBCE*, *DNAJA3*, *CALCA*, *SLC22A18*, *DCAF11*, *ACTL8*, *SH3TC1*, *GRM4*, and *DHRS2*. Finally, four other genes, *TFCP2L1*, *FOXL2*, *LY6E*, and *EXOC3L1*, were differentially expressed and downregulated in MRuc5NPC, but, to date, no firm association with neuronal development has been inferred.

### 3.7. Protein–Protein Interactions and Hub Gene Analysis for Human NPCs Exposed to EVs from Human TBs or iPSCs Vs. Non-Exposed NPCs

**Figure 7:**
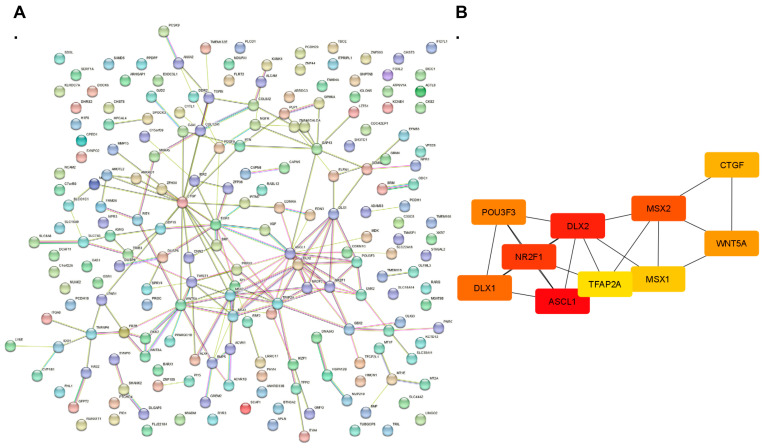
STRING and hub gene analyses for proteins differentially expressed between control NPCs vs. TB EVs treated with NPCs. (**A**) Protein–protein interactions (PPI) were determined by STRING analysis. (**B**) The PPI files generated with STRING were imported into the cytoHubba app [67] in Cytoscape [59] to determine the top 10 hub proteins. Within this program, hub proteins were determined with MCC analysis as recommended [67].

We next considered the potential interactions of proteins encoded by the affected transcripts, including those that might be co-expressed together and those protein-encoding genes that might be at the center or focal point of such interactions, otherwise considered hub genes. The STRING analysis for the protein interactions of those transcripts differentially expressed in NPCs treated with TB EVs relative to control NPCs shows one main cluster in the center. The importation of the STRING results into the cytoHubba program [67] reveals that the top 10 hub genes, i.e., those at the center of the interactions, were *ASCL1*, *NR2F*, *DLX2*, *POU3F3*, *DLX1*, *MSX2*, *CTGF*, *WNT5A*, *MSX1*, and *TFAP2A*, all of which except *CTGF* and *WNT5A*, encode transcription factors (Figure 7).

**Figure 8:**
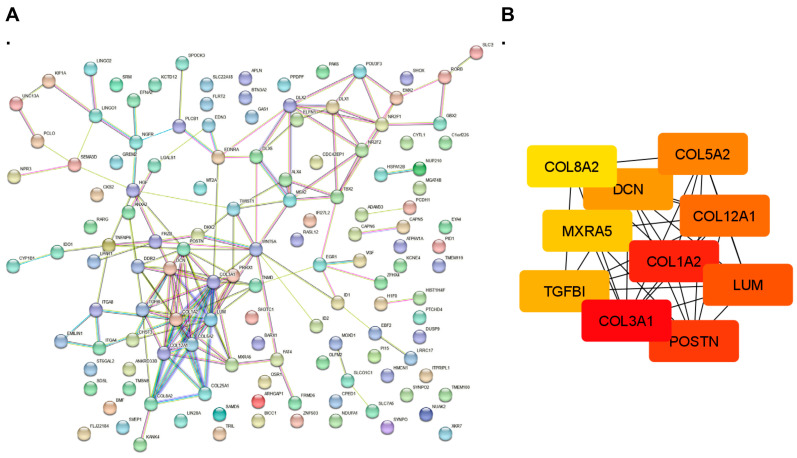
STRING and hub gene analyses for proteins differentially expressed between control NPCs vs. iPSC EVs treated with NPCs. (**A**) Protein–protein interactions (PPI) were determined by STRING analysis. (**B**) The PPI.files generated with STRING were imported into the cytoHubba (Version 0.1) app [67] in Cytoscape [59] to determine the top 10 hub proteins. Within this program, hub proteins were determined with MCC analysis as recommended [67].

The STRING analysis of differentially regulated genes in NPCs treated with iPSC EVs compared to control NPCs reveals two main clusters. All but one (*TGFB1*) of the top ten hub genes for this comparison consist of genes associated with extracellular matrix modeling (*COL1A2*, *COL3A1*, *COL8A2*, *COL5A2*, *COL12A1*, *POSTN, LUM, DCN*, and *MXRA5*, Figure 8). Even TGFB is generally complexed within the ECM where it is activated [80].

### 3.8. Pathways Predicted to Be Affected by the Exposure of Human NPCs to EVs Derived from Human TBs or iPSCs

**Figure 9:**
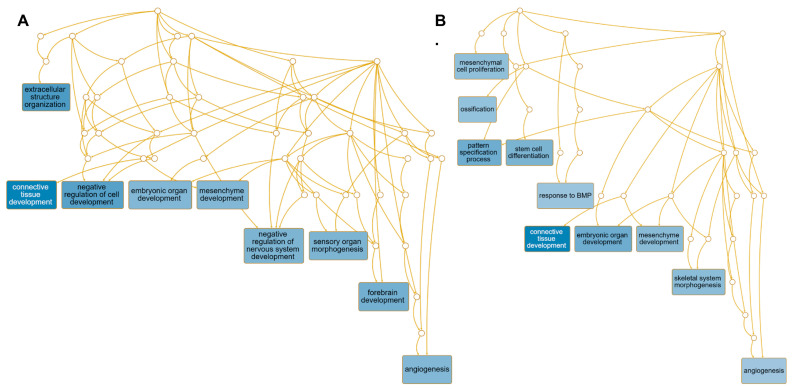
Gene ontology biological process (GO BP) and molecular function (GO MF) pathways are predicted to be affected based on differentially expressed genes. This was determined by using the WEB-based GEne SeT AnaLysis Toolkit (WebGestalt) 2019 version online program. (**A**) Control NPCs vs. TB EVs treated with NPCs. (**B**) Control NPCs vs. iPSC EVs.

The primary pathways predicted to be affected in NPCs treated with TB EVs relative to control NPCs include those associated with regulating forebrain development, the negative regulation of the nervous system development, extracellular structure organization, embryonic organ development, sensory organ morphogenesis, connective tissue/mesenchyme development, and angiogenesis (Figure 9).

The main pathways predicted to be affected in NPCs treated with iPSC EVs include mesenchymal cell proliferation, the pattern specification process, stem cell differentiation, the response to BMP (bone morphogenetic protein), connective tissue development, embryonic organ development, mesenchyme development, and angiogenesis (Figure 9).

### 3.9. Brain Enrichment for Transcripts Altered in Human NPCs Exposed to EVs Derived from Human TBs or iPSCs

**Figure 10:**
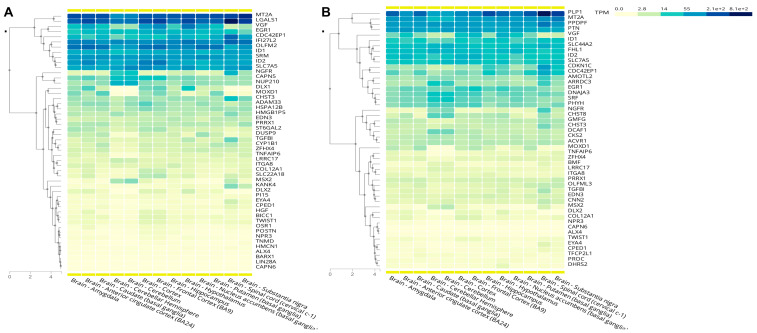
Brain-specific gene enrichment analysis for differentially expressed genes was determined by the GTEx Portal (API V2) [69]. This was performed for the top 50 differentially expressed genes in each of the comparisons and by searching all brain regions in this database. (**A**) Control NPCs vs. TB EVs treated with NPCs. (**B**) Control NPCs vs. iPSCs treated with NPCs.

The GTEx Portal (API V2) was used to examine the top differentially expressed (up- and downregulated) genes and their expression pattern in the various brain regions of humans. For NPCs treated with TB EVs, several of the genes are enriched in various brain regions, including the amygdala, basal ganglia, cerebellum, cortex, anterior cingulate cortex, frontal cortex, hippocampus, hypothalamus, nucleus accumbens, and substantia nigra (Figure 10).

Example transcripts include the following: *MT2A*, *LGALS1*, *VGF*, *EGR1*, *CDC2EP1*, *IFI27L2*, *OLFM2*, *ID1*, *SRM*, *ID2*, *SLC7A5*, *NGFR*, *CAPN5*, and *NUP210*. For the top differentially expressed genes in NPCs treated with iPSC EVs, several transcripts were also enriched in the same brain regions detailed above, but the transcripts affected by iPSC EVs appeared to be less abundant than those affected by TB EVs (Figure 10). Examples include *PLP1*, *MT2A*, *PPDPF*, *PTN*, *VGF*, *ID1*, *SLC44A2*, *FHL1*, *ID2*, *SLC7A5*, and *CDKN1C.*

### 3.10. Linkages Between Differentially Expressed miRs and lncRNAs in Human TB-Derived EVs and Transcriptomic Changes in Human NPCs Treated with Human TB-Derived EVs

**Figure 11:**
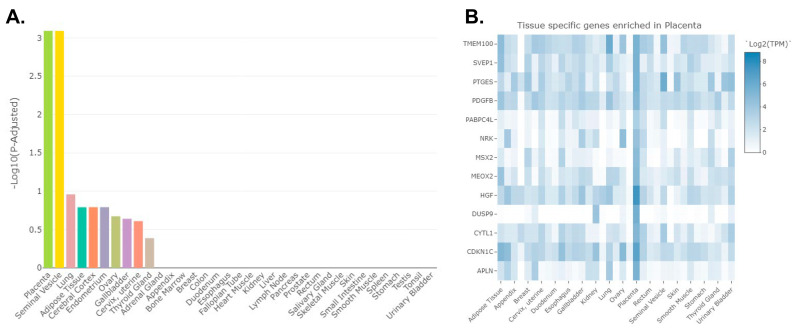
Tissue enrichment analysis based on the TissueEnrich program [46] with the 185 differentially expressed transcripts in human NPCs treated with TB-derived EVs that intersect with miR and lncRNA changes within EVs. (**A**) These transcripts are primarily associated with the placenta, followed by seminal vesicles, long, adipose tissue, the cerebral cortex, endometrium, ovary, gallbladder, cervix/uterine, and thyroid gland. (**B**) Heat map analysis reveals that the transcripts that are abundant in the placenta include *TMEM100*, *SVEP1*, *PTGES*, *PDGFB*, *PABPC4L*, *NRK*, *MSX2*, *MEOX2*, *HGF*, *DUSP9*, *CYTL1*, *CDKN1C*, and *APLN*.

The NcPath program (Used on 20 September 2024) [54], which provides KEGG [55] pathway associations, was used to reveal possible interactions between miR and lncRNA changes in TB-derived EVs and transcriptomic changes induced in NPCs treated with these EVs. This approach established linkages between 26 miRs, 185 mRNA transcripts, and 786 lncRNAs (Supplementary File S7). The 185 differentially expressed transcripts in human NPCs treated with TB-derived EVs that intersected with miR and lncRNA changes within EVs derived from TB cells were imported into the TissueEnrich program [43]. This analysis revealed that these transcripts are primarily associated with the placenta followed by seminal vesicles, long, adipose tissue, the cerebral cortex, endometrium, ovary, gallbladder, cervix/uterine, and thyroid gland (Figure 11).

Heat map analysis reveals that the transcripts that are abundant in the placenta include *TMEM100*, *SVEP1*, *PTGES*, *PDGFB*, *PABPC4L*, *NRK*, *MSX2*, *MEOX2*, *HGF*, *DUSP9*, *CYTL1*, *CDKN1C*, and *APLN* (Figure 11).

Pathways that are affected by the 185 differentially expressed transcripts in human NPCs treated with TB-derived EVs that intersect with miR and lncRNA changes within EVs derived from TB cells are shown in Supplementary Figure S7 and listed in Supplementary File S7. These pathways include neurotrophin, estrogen, GnRH, and oxytocin signaling, as well as pathways associated with axon guidance. The overlap between these pathways and miRs, lncRNAs, and transcripts within these pathways is shown in Supplementary Figure S8 (a low-magnification view that lists all miRs, lncRNAs, and transcripts), and Supplementary Figure S9 (a higher magnification view focused on the pathways above).

## 4. Discussion

The goal of the research presented here has been to determine whether there is evidence to support the existence of a placenta–fetal brain axis in humans. Our hypothesis was that non-coding RNAs, especially miRs and lncRNAs, present in TB-derived EVs have the potential to influence gene expression in neuronal cell precursors in such a manner that it might portend a shift towards a more differentiated or, at the very least, altered function. To test this hypothesis, we isolated EVs from TBs that had been differentiated from a human iPSC cell line, assessed their small RNA content, and evaluated their ability to alter the transcriptome of cultured NPCs. As controls, we conducted the same experiments with EVs from undifferentiated iPSCs, which lack a TB phenotype [34]. One limitation of this study is that the method employed for directing differentiation, the BAP procedure, produces TB cells that include two forms of syncytioTB: a heterogeneous extra-villous TB population and various forms of cytoTB, i.e., a mixture of differentiated and undifferentiated TB cell types [81]. Accordingly, it is not possible to infer the main TB cell types that contribute to the EVs inducing changes in the NPCs. A second limitation of this work is that the variation in the non-coding RNA content between the iPSCs and TBs derived from them is not much greater than the variation occurring within the two groups themselves, which is particularly true for the iPSCs. In part, this may be due to the tendency of the iPSC to differentiate spontaneously but also due to the various subpopulations of cells that have been observed to exist in pluripotent cell cultures, which are notoriously metastable [82]. There is overlap between the EVs from TBs and those from the iPSCs in their content of various non-coding RNAs, which is also perhaps possible, as the differentiation procedure was only for a few days, and the TB cultures likely contain populations of relatively immature progenitors not far-removed in transcriptome profiles from the parental iPSCs.

The outcome of these studies was that both kinds of EVs, i.e., those from iPSCs differentiated to TBs and the controls differentiated from undifferentiated iPSCs, contained a wide range of small RNAs, including miRs and lncRNAs, but that there were significant compositional differences between the two. The biggest differences were in the lncRNA composition, with over 6000 components identified as differentially expressed in the EVs from TB, of which about two-thirds were upregulated. The large number of changes observed in lncRNAs is perhaps not surprising, as their heterogeneity exceeds that of mRNAs in many stable cell lines, including those that are pluripotent [83,84]. With few exceptions, little is still known about the function and control of expression for lncRNAs. It was once thought that they were anomalies and possibly transcriptional background noise. However, it is becoming clear that they may have crucial functions. In the placenta, they have been proposed to have roles in regulating cell proliferation, invasion, and apoptosis [85].

Differences in miR content were far fewer than those for lncRNAs (Table 1) and possibly easier to interpret. Among the 20 most upregulated miRs in the EVs from TB, a few, for example, *hsa-miR-0149-3p* and *hsa-302a-5p*, are also enriched in the human brain, especially in the cerebral cortex and have been inferred to be involved in nervous system development (Figure 2). Because miRs can control gene expression through their abilities to interact with protein-coding mRNAs, we established likely targets for some of those that are enriched in TB-derived EVs. Interestingly, the primary mRNA targets for *hsa-miR-0149-3p*, like the miR itself, are enriched almost exclusively in the cerebral cortex (Figure 3), while those for *hsa-302a-5p* have a broader tissue distribution but one that includes the cerebral cortex. By contrast, another upregulated miR, *hsa-miR-395*, is inferred to target transcripts abundant in the cervix and uterus, i.e., maternal genital tract tissue (Figure 3) rather than the brain. Conceivably, the EVs released by human TBs are directed towards several recipient cell types, some maternal, others fetal, and not just progenitors of the developing brain. Such heterogeneity should not be surprising considering that BAP-directed differentiation gives rise to more than one TB type, including synctioTB and extravillous TB [39,81,86,87] (each of which likely produces its own spectrum of EVs).

Our next experiments revealed that human NPCs (MRuc5NPC) are able to internalize EVs produced by both the control cells (human iPSC; MRuc5i) and TB cells derived from iPSC in a time-dependent and in what appeared to be relatively efficient manner (Figure 5). Once internalized, the marker dye associated with the EVs assumes a perinuclear localization, consistent with EV uptake occurring by endocytosis. Whether EV contents are subsequently transferred to the nucleus, where they might affect gene expression directly, or whether they operate post-transcriptionally on processes occurring in the cytoplasm is unknown. What is evident is that the uptake of the EVs is followed by changes in the transcriptome of the NPC, but what is unclear is whether there is any specificity to this process, such as the selective uptake of only a certain kind of EV by a particular target cell type.

Transcriptome analysis revealed that the treatment of NPCs with EVs provided a switch in gene signature patterns no matter the source of the EVs, as evidenced in the PCA and volcano plots (Figure 6). Moreover, both kinds of EVs, i.e., those from iPSCs and TBs, tended to influence the transcript levels of many genes commonly associated with neural tissues. However, TB-derived EVs provided changes in a greater number of neural-associated transcripts biased towards pathways predicted to regulate forebrain development, the negative regulation of the nervous system development, and sensory organ morphogenesis. In contrast, EVs from iPSCs appeared more likely to direct NPCs towards mesenchymal development and proliferation, connective tissue development, and angiogenesis, and an overall transcriptional phenotype resembling that of glial cells rather than neurons. This observation is perhaps not surprising, as cortical neurons and glia are known to be generated from NPCs during the development of the cerebral cortical issue, with the number and type of differentiating cells dependent upon the biochemical inputs [88]. On the other hand, the very exposure to EVs, no matter what their origin, may lead to some common responses in NPCs.

Our findings with his human in vitro model are quite similar to those we observed when mouse NPCs were exposed to EVs from mouse trophoblast stem cells (TSCs) and TSCs differentiated to parietal trophoblast giant cells, which, like human extravillous trophoblast cells, are in direct proximity to maternal decidual cells [32]. In those studies, the miRs and small nucleolar (sno)RNA profiles varied based on the EV source, with snoRNA exclusively upregulated in EVs from pTGC. The primary inferred targets of the miRs from both pTGCs and TSCs were again transcripts enriched in the fetal brain. As with human cells, mouse NPCs rapidly internalized EVs, resulting in major transcriptome changes. Differentially expressed genes were again enriched in neural tissues. Finally, differentially regulated transcripts in NPC that had been exposed to EVs had functions linked to neuronal development.

These initial studies were limited in scope to testing for gene expression changes in human NPCs following 24 h of treatment with EVs from human TBs or iPSCs. Future studies need to be performed to determine whether temporal effects exist following such exposures. Additionally, it will be of interest to determine whether specific miRNAs or other ncRNAs within the EVs are driving the transcriptomic changes in NPCs. EVs could be engineered to include labeled forms of miRs identified to be enriched in TB EVs, such as for *hsa-miR0149-3p*, *hsa-miR-302a-5p*, and *hsa-miR-935*. The amounts of these miRs within the NPC could then be determined, and RNAseq analysis performed to examine whether one or more of these miRs affect transcripts within NPCs. Further work should assess whether differentiated neural cell types show contrasting responses to EVs derived from TB and iPSC relative to each other and the undifferentiated NPCs evaluated herein.

In conclusion, these studies reveal that among the cargo carried by human TB-derived EVs are lncRNA and miRs. Although we are not able to comment on the roles of the former, many of the miRs are abundant in neural issues, including the cerebral cortex. These studies also demonstrate that human TB- and iPSC-derived EVs can be internalized by human NPCs. This uptake in EVs leads to changes in the NPC transcriptome. In the case of EVs from TB cells, there is an upregulation of transcripts particularly associated with forebrain formation and neurogenesis, suggesting that such EVs have a role in early fetal brain development. The EVs from iPSC appear to favor a switch towards a glial cell phenotype. The results support that TB-derived EVs are key mediators in a functional placenta–brain axis in humans.

## Figures and Tables

**Table 1:** Top miR expression differences between EVs derived from TB cells vs. iPS cells.

miR	log2 FoldChange	Fold Change	*p*-Value	Adjusted *p* Value	Directionality TB EV vs. iPS EV
hsa-miR-149-3p	10.05171607	1061.373023	0.00048491	0.031022969	Up
hsa-miR-4788	−11.94158699	0.000254228	0.00048855	0.031022969	Down
hsa-miR-19b-3p	9.838790506	915.7376353	0.00109125	0.046196118	Up
hsa-miR-23a-3p/23b-3p	8.684175054	411.3364341	0.00149931	0.04623086	Up
hsa-miR-574-3p	9.124625271	558.1949534	0.00182011	0.04623086	Up
hsa-miR-92a-3p	7.299724875	157.5564357	0.00249319	0.052772551	Up
hsa-miR-151a-3p	6.448213714	87.31839541	0.00772971	0.073415825	Up
hsa-miR-19a-3p	8.847565464	460.6622169	0.00924924	0.073415825	Up
hsa-miR-21-5p	8.150478226	284.1439576	0.00627482	0.073415825	Up
hsa-miR-24-3p	6.923785137	121.4135087	0.00636052	0.073415825	Up
hsa-miR-28-3p	7.657611066	201.9159497	0.00911805	0.073415825	Up
hsa-miR-302a-5p	7.789624074	221.2638685	0.00759028	0.073415825	Up
hsa-miR-335-5p	8.214290328	296.9940726	0.00540517	0.073415825	Up
hsa-miR-4497	−8.622843036	0.002536679	0.00910335	0.073415825	Down
hsa-miR-489-3p	8.278285493	310.464713	0.00588676	0.073415825	Up

## Data Availability

Small RNA sequencing data were deposited in the Gene Expression Omnibus under accession ID GSE271631. RNA sequencing data were deposited in the Gene Expression Omnibus under accession ID GSE226979.

## Supplementary Materials

The following supporting information can be downloaded at: https://www.mdpi.com/article/10.3390/cells13221867/s1, Figure S1: Small RNAseq results for EVs from TB and iPSC; Figure S2: Heatmaps for differentially expressed small RNA in EVs from TB vs. EVs from iPSC; Figure S3: Chromosomal distribution analysis for differentially expressed miRs and lncRNAs between EVs derived from TB vs. iPSC; Figure S4: TissueEnrich program [46] analysis for other miRs upregulated in TB EVs vs. iPSC EVs; Figure S5: Pathway analysis for target mRNAs based on differentially expressed miRs; Figure S6: Venn diagram comparison of differentially expressed genes for Control NPC vs. TB EVs NPC and Control NPC vs. iPSC EVs; Figure S7: KEGG pathways affected by the intersection of differentially expressed miRs and lncRNAs in human TB cells and transcripts differentially expressed by human NPC treated with EVs; Figure S8: KEGG pathways of interest, including neurotrophin signaling pathway, estrogen signaling path-way, GnRH signaling pathway, oxytocin signaling pathway, axon guidance, and overlap between these pathways and miRs, lncRNAs, and transcripts within each of these pathways; Figure S9: Higher magnification image of select KEGG pathways of interest, and miRs, lncRNAs, and tran-scripts within each of these pathways. Table S1: Small RNAseq results on reads for small RNAs contained within the EVs isolated from each of the samples, and the average number for the different types of small RNA; Table S2: Alignment details for RNA-Seq dataset from human neural progenitor cells (NPC). File S1: To analyze miR differences within EVs isolated from TB vs. iPSC, the miRge3 program [43] followed by DESeq2 was used [71]; File S2: Analysis of hsa-miR0149-3p (the top upregulated miR in EVs derived from TB cells) with the miRPathDB reveals that the primary GO biological processes affected by this miR include nervous system development, anatomical structure, morphogenesis, neurogenesis, generation of neurons, and positive regulation of gene expression; File S3: Long non-coding (lnc) RNA and other small RNA differences were analyzed, we utilized the human reference genome (GRCh38) from GENCODE [72] and GFF3 files from the RNACentral [47] database, and DESeq2 was used to identify differentially expressed lncRNAs with an adjusted *p*-value threshold of less than 0.05 and a log2 fold change of 2, yielding 6794 lncRNAs; File S4: Analysis of differentially expressed miRs with the https://mirdb.org/ database [71,72] to predict the target transcripts of for hsa-miR0149-3p, hsa-miR-302a-5p, and hsa-miR-935. Since miRs can regulate gene expression by pairing with particular mRNA, usually in their 3^/^-termini, the https://mirdb.org/ database [71,72] was then used to predict the target transcripts of for hsa-miR0149-3p, hsa-miR-302a-5p, and hsa-miR-935 that are upregulated in EVs from TB cells and are enriched in the brain and nervous tissues; File S5. ClueGO [47], which is a Cytoscape plug-in, was used to determine gene ontology and pathways that are predicted to be affected by mRNAs targeted by miRs differentially expressed by miRs in TB EVs vs. iPSC EVs; File S6: All the differentially expressed genes in NPC treated with EVs from TB or iPSC or not treated with any EVs (control NPC); File S7: To understand interactions between miRs and lncRNA changes in TB-derived EVs and transcriptomic changes in NPC treated with these structures the NcPath [54] database, which provides KEGG [55] pathway associations, was used. Video S1. 3D confocal video of human NPC with internalized human TB EVs 40 min post-treatment; Video S2. 3D confocal video of human NPC with internalized human iPSC EVs 40 min post-treatment.
