# Supplementary material for: Small and Long Non-Coding RNA Analysis for Human Trophoblast-Derived Extracellular Vesicles and Their Effect on the Transcriptome Profile of Human Neural Progenitor Cells
Source: Cells. 2024 Nov 11;13(22):1867. doi: 10.3390/cells13221867 (PMC11593255; doi:10.3390/cells13221867)
Supplement: Supplementary file 1 [file cells-13-01867-s001.zip › cells-3259524-supplementary.pdf]

cells

MDPI

Supplementary Materials

Small and Long Non-Coding RNA Analysis for Human Trophoblast-Derived Extracellular Vesicles and Their Effect on the Transcriptome Profile of Human Neural Progenitor Cells

Jessica A. Kinkade [1,2]†, Pallav Singh [3,†], Mohit Verma [2], Teka Khan [5], Toshihiko Ezashi [2,4,5], Nathan J. Bivens [6], R. Michael Roberts [5,7], Trupti Joshi [2,3,8,*] and Cheryl S. Rosenfeld [1,3,9,10,*]

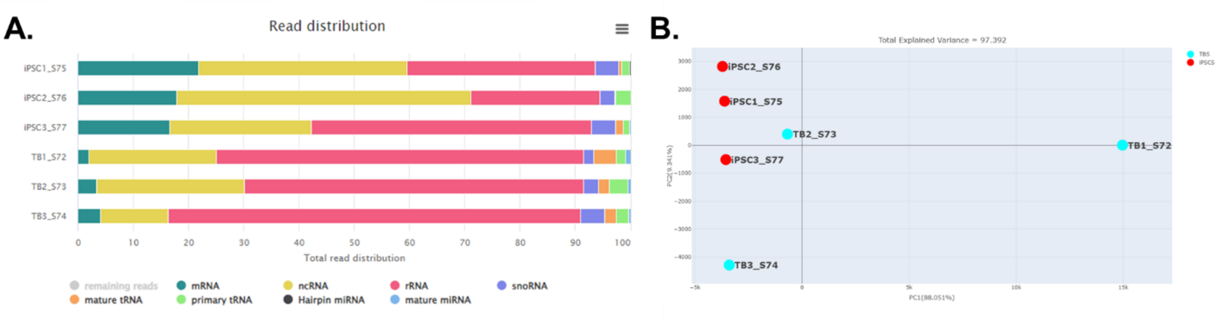

Supplementary Figure S1. Small RNAseq results for EVs from TB and iPSC. A) Breakdown for types of small RNA identified in EVs for each of the TB and iPSC cell replicates. B) A 2D PCA plot based on all small RNA reveals there is no clear clustering between EVs derived from iPSC vs. those derived from TB cells (PERMANOVA value = 0.1 for both miR and lncRNA).

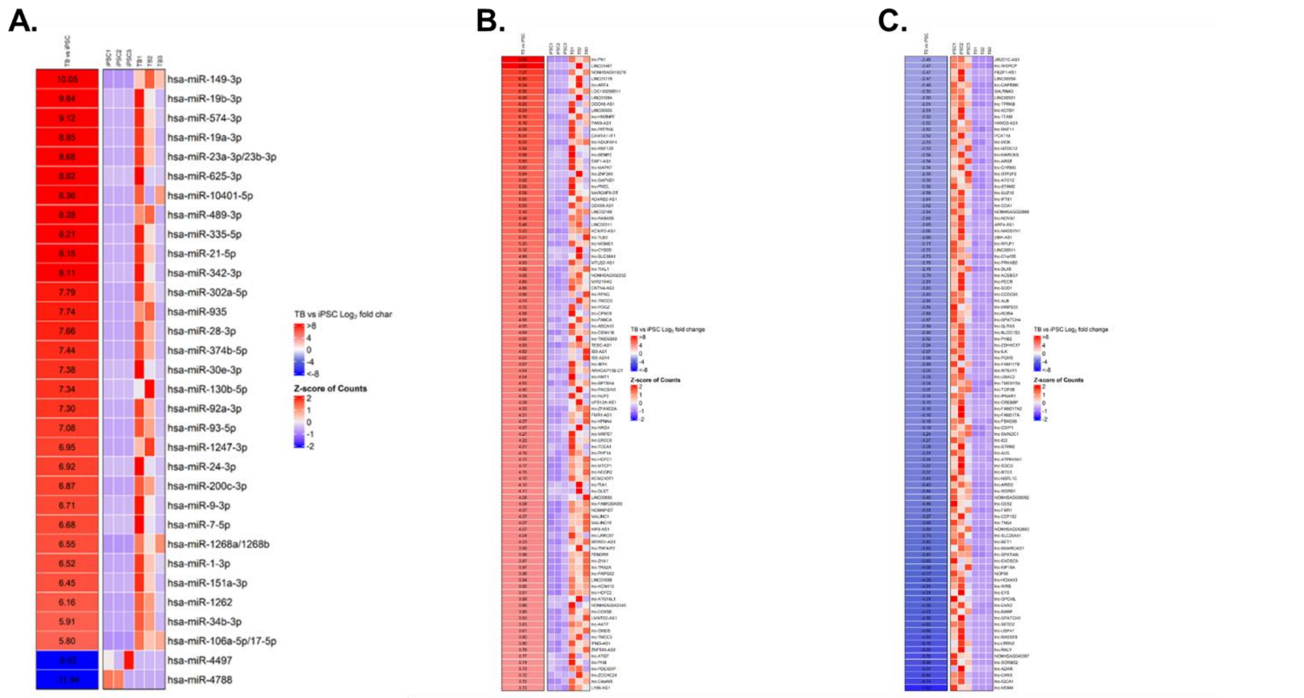

Supplementary Figure S2. Heatmaps for differentially expressed small RNA in EVs from TB vs. EVs from iPSC. A) Heatmap for all miRs that are differentially expressed in EVs from TB vs. EVs from iPSC. B) Top 100 long ncRNA upregulated in EVs from TB vs. EVs from iPSC. C) Heatmap for top 100 downregulated lncRNA in EVs from TB vs. EVs from iPSC.

Cells **2024**, *13*, 1867. https://doi.org/10.3390/cells13221867 www.mdpi.com/journal/cells

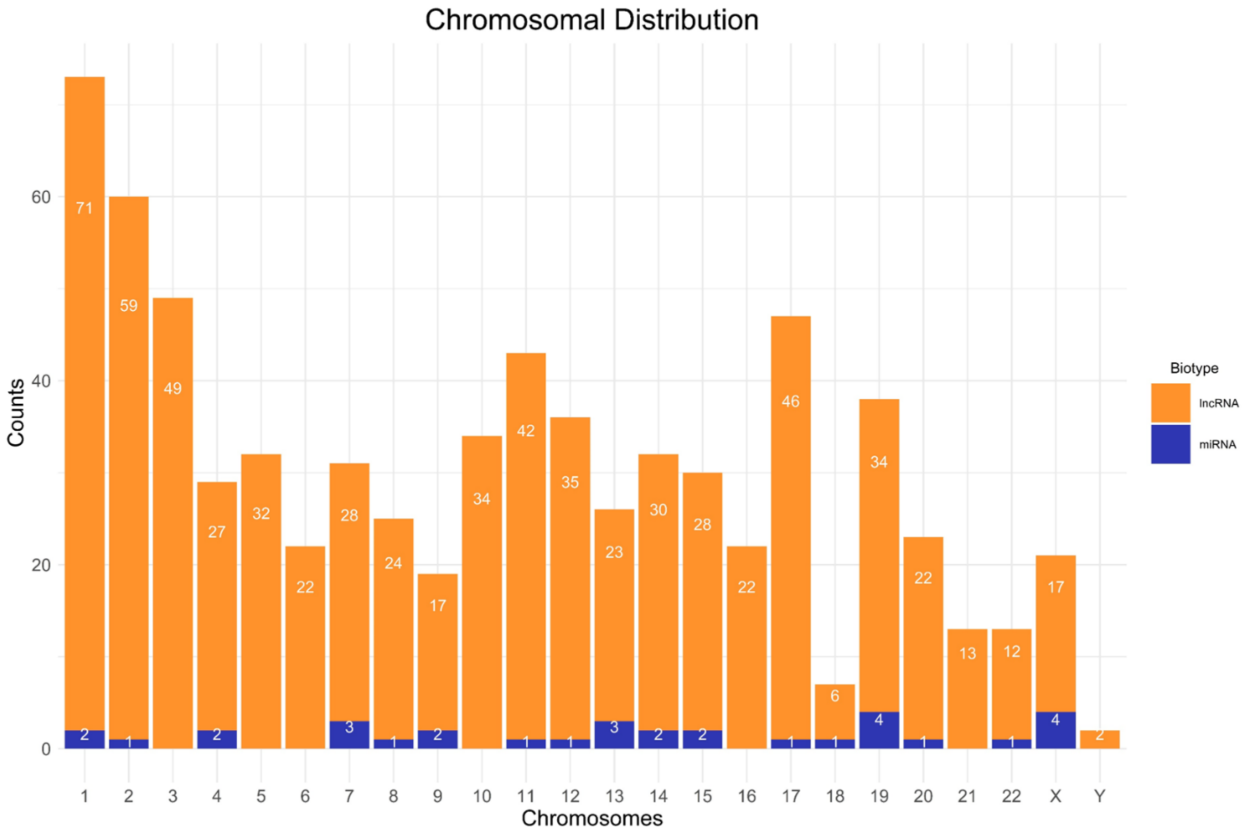

Supplementary Figure S3. Chromosomal distribution analysis for differentially expressed miRs and lncRNAs between EVs derived from TB vs. iPSC. Analysis of the chromosomal distribution for the differentially expressed miR and lncRNA revealed that most of the differentially expressed ones were on Chromosome 1, followed by Chromosomes 2, 3, 17, 11, 12, and then 19.

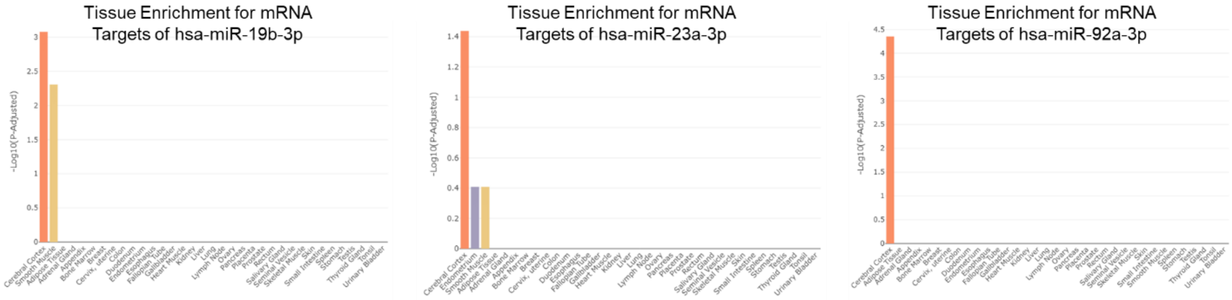

Supplementary Figure S4. TissueEnrich program [1] analysis for other miRs upregulated in TB EVs vs. iPSC EVs. For hsa-miR-19b-3p, target mRNAs are enriched in the cerebral cortex and skeletal muscle. For hsa-miR-23a-3p, target mRNAs are enriched in the cerebral cortex, endometrium, and smooth muscle (hsa-miR-23a-3p), and target mRNAs for hsa-miR-92a-3p) are enriched in the cerebral cortex.

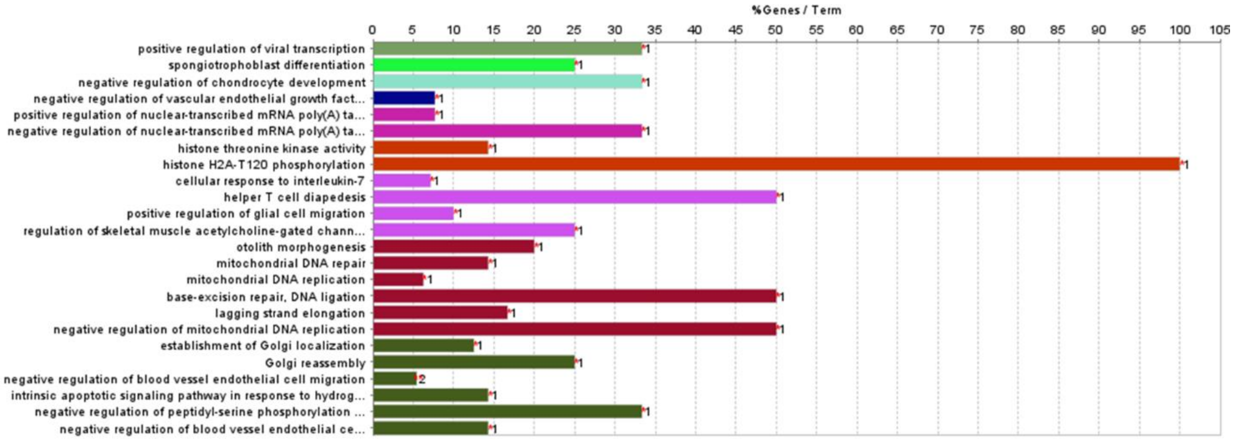

Supplementary Figure S5. Pathway analysis for target mRNAs based on differentially expressed miRs. The consideration of all mRNA targets for the differentially expressed miRs reveals that they are associated with the following pathways: the positive regulation of viral transcription, spongiotrophoblast differentiation, histone H2A T120 phosphorylation, mitochondrial DNA repair and migration, DN replication and ligation, and T helper cell responses to list a few examples.

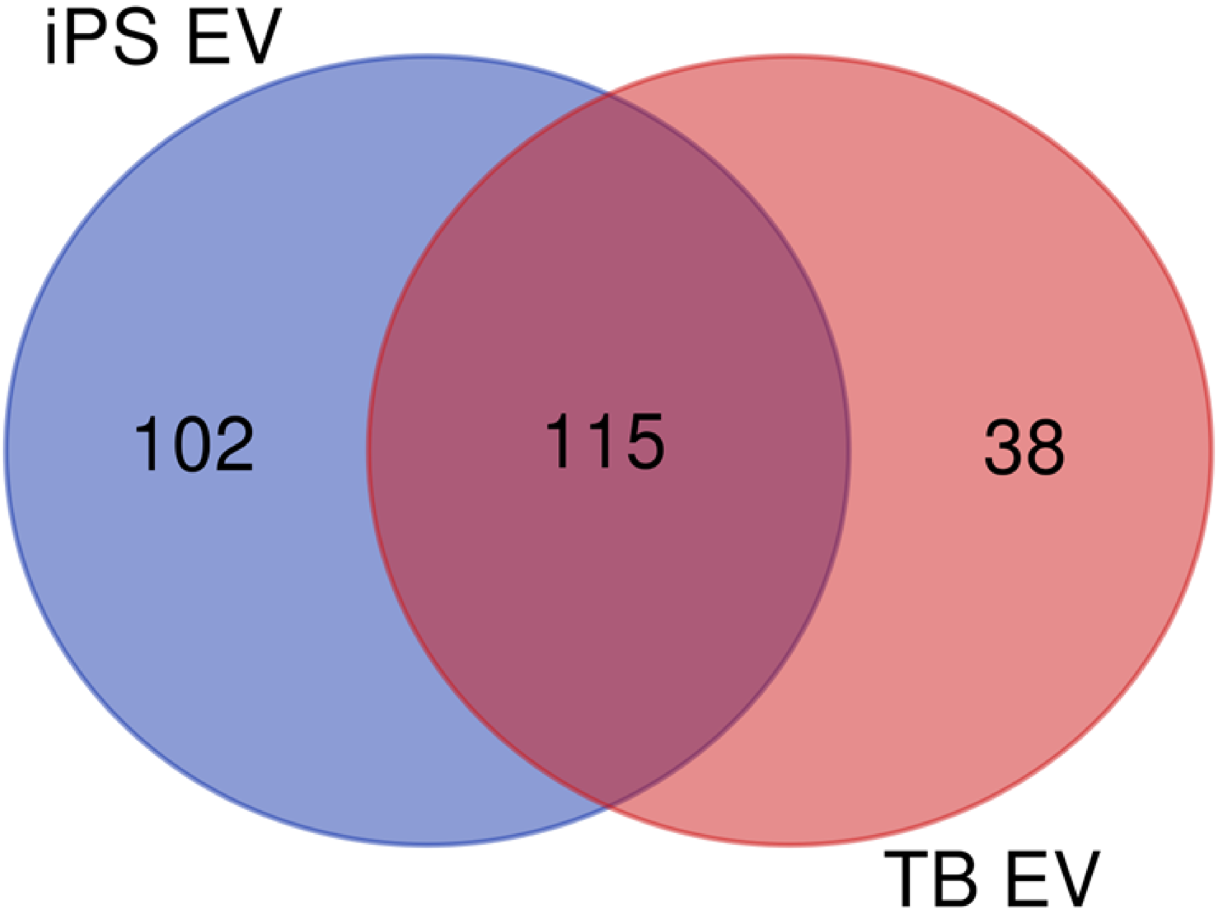

Supplementary Figure S6. Venn diagram comparison of differentially expressed genes for Control NPC vs. TB EVs NPC and Control NPC vs. iPSC EVs.

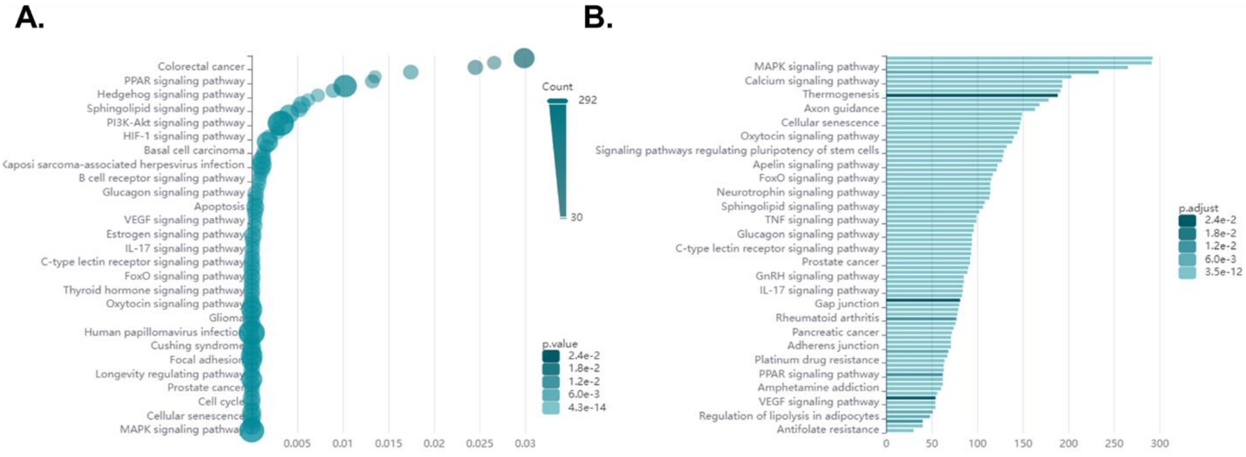

Supplementary Figure S7. KEGG pathways affected by the intersection of differentially expressed miRs and lncRNAs in human TB cells and transcripts differentially expressed by human NPC treated with EVs. A) KEGG pathways impacted by this intersection based on the p-value. The size of the dots shows the total number of genes in each pathway. B) KEGG pathways affected by this intersection based on the total number of genes in that pathway as ranked in descending order for the left graph.

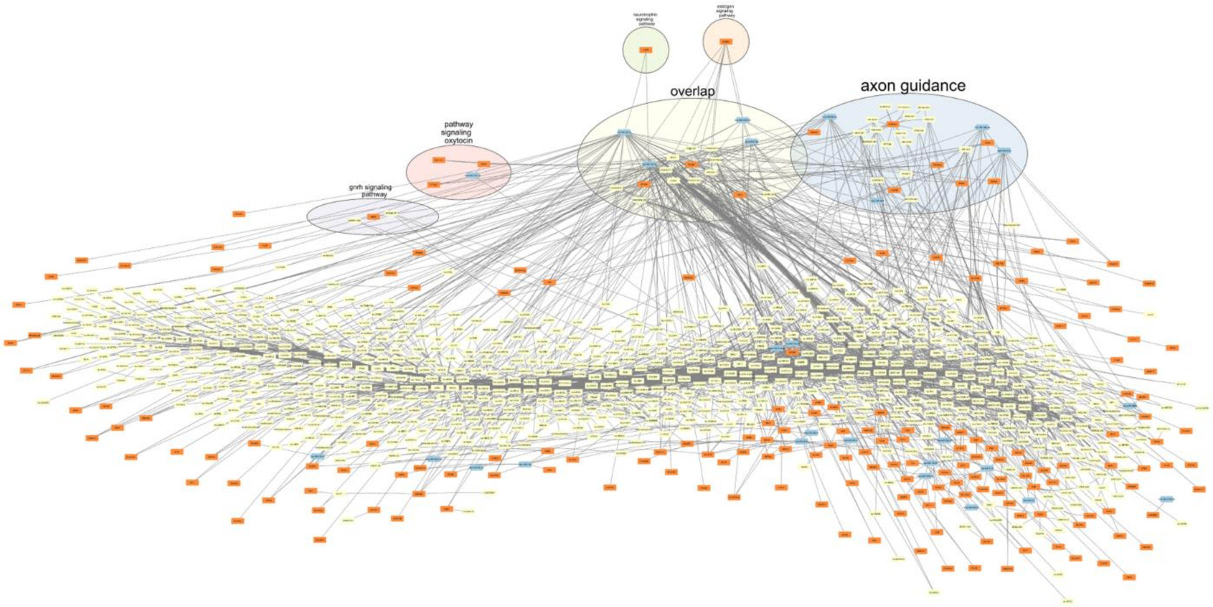

Supplementary Figure S8. KEGG pathways of interest, including the neurotrophin signaling pathway, estrogen signaling pathway, GnRH signaling pathway, oxytocin signaling pathway, axon guidance, and the overlap between these pathways and miRs, lncRNAs, and transcripts within each of these pathways.

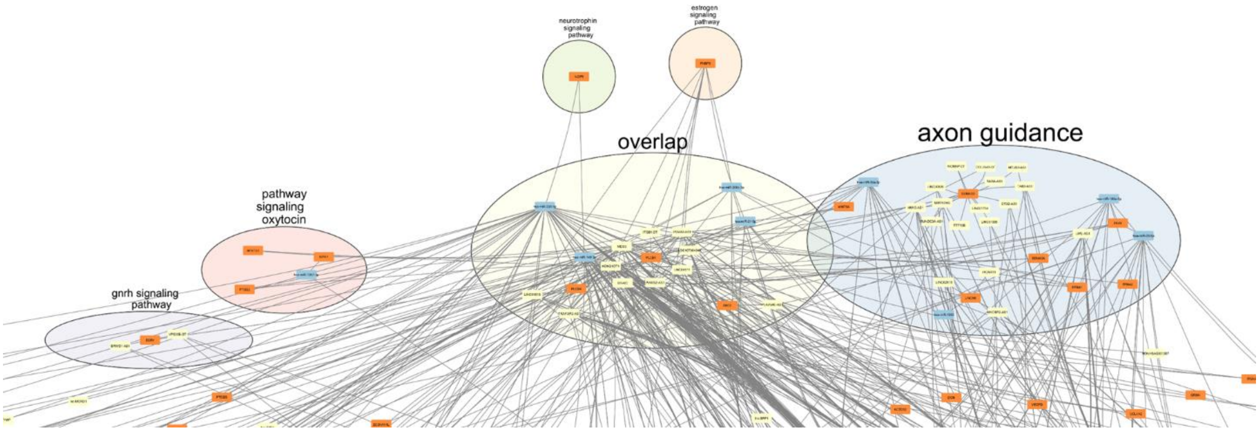

Supplementary Figure S9. Higher magnification image of select KEGG pathways of interest and the miRs, lncRNAs, and transcripts within each of these pathways.

Supplementary Table S1. Small RNAseq results from the reads of small RNAs contained within the EVs isolated from each of the samples and the average number for the different types of small RNAs. iPS= induced pluripotent stem cells, and TB= trophoblast cells.

Sample name	Total Input Reads	Trimmed Reads (all)	Trimmed Reads (unique)	Total miR*	Filtered miR**	Unique miR***	Hairpin miR	Primary tRNA	Mature tRNA	snoRNA	rRNA	ncRNA
**iPSC1_S75**	30676740	29550947	5327865	233538	6127	38	3238	151460	61520	426986	3481392	3847838
**iPSC2_S76**	23780185	22837302	3433930	62037	2142	19	1502	275595	15802	270246	2387420	5446821
**iPSC3_S77**	22860682	21777831	4667322	113255	12453	59	3073	79349	110085	318446	3811937	1925531
**Average for iPSC**	25772535.67	24722027	4476372	136276.7	6907.333	38.66667	2604.333	168801.3	62469	338559.3	3226916	3740063
**TB1_S72**	27206477	26196098	4773887	165997	58539	235	3887	127568	296314	135817	4921655	1705279
**TB2_S73**	19384340	18560460	3050140	110699	22295	92	2002	200120	116566	162541	3659810	1600812
**TB3_S74**	22943581	21830016	4286294	90803	21724	126	6542	220216	203248	427875	7224940	1190562
**Average for TB**	23178132.67	22195525	4036774	122499.7	34186	151	4143.667	182634.7	205376	242077.7	5268802	1498884

*The total miR refers to the number of reads found in the samples that fit the definition of miRNA with the lengths and canonical definitions that are defined within the miRge program [2]. These criteria are stringent and attempt to miR only.

**Once the total miRNA reads are determined, the miRge program collapses these reads down based on their similarity in the sample, which is shown in the filtered miR reads. The number in the filtered miRNA column reads refers to the number of miRs, while the count of these miRs is tabulated in the back, hence the need to remove identical reads.

*** Unique miRNA refers to miR identified by alignment and annotated within the filtered miRNA reads and further removes miRs that have no assignment to an annotation or which can be aggregated further based on a less stringent parameter. These parameters are less stringent because miRs are small, and any differences in the read will be considered high in terms of the percentage of change compared to the total number of base pairs in the miRNA.

Supplementary Table S2. Alignment details for the RNA-Seq dataset from human neural progenitor cells (NPCs).

Sample Number	Treatment	# of Total Paired-End Reads	% Alignment	# of Total Mapped Paired-End Reads
NPC_Control_Sample1_S24	Control	28679269	93.1	26693390
NPC_Control_Sample2_S25	Control	25894179	93.7	24266933
NPC_Control_Sample3_S29	Control	20644462	93.1	19217563
NPC_Control_Sample4_S28	Control	31045469	93.6	29053279
NPC_TB_EV_Sample1_S26	TB-EV	43300141	93.1	40301525
NPC_TB_EV_Sample2_S23	TB-EV	37399418	93.5	34953388
NPC_TB_EV_Sample3_S22	TB-EV	44783306	94.0	42110795
NPC_TB_EV_Sample4_S21	TB-EV	38872267	93.6	36394629
NPC_iPS_EV_Sample1_S20	iPS-EV	41683924	93.3	38882617
NPC_iPS_EV_Sample2_S19	iPS-EV	34525558	94.4	32605230
NPC_iPS_EV_Sample3_S18	iPS-EV	34890423	93.8	32734027
NPC_iPS_EV_Sample4_S27	iPS-EV	37580571	92.8	34858380
**Average**		**34941582**	**93.5**	**32672646**

Supplementary File S1. To analyze miR differences within EVs isolated from TB vs. iPSC, the miRge3 program [2] followed by DESeq2 was used [3]. Based on a p-value threshold of 0.05 and a log2 fold change of 2, 32 differentially expressed miRs were identified to be differentially expressed, and 5 miRs were differentially expressed based on a p-adjusted value (FDR) and log2 fold change of 2.

Supplementary File S2. The analysis of hsa-miR0149-3p (the top upregulated miR in EVs derived from TB cells) with the miRPathDB reveals that the primary GO biological processes affected by this miR include nervous system development, anatomical structure, morphogenesis, neurogenesis, the generation of neurons, and positive regulation of gene expression. This file lists other pathways predicted to be affected by the upregulation of hsa-miR0149-3p.

Supplementary File S3. Long non-coding (lnc) RNA and other small RNA differences were analyzed; we utilized the human reference genome (GRCh38) from GENCODE [4] and GFF3 files from the RNACentral [5] database, and DESeq2 was used to identify differentially expressed lncRNAs with an adjusted p-value threshold of less than 0.05 and a log2 fold change of 2, yielding 6,794 lncRNAs.

Supplementary File S4. Analysis of differentially expressed miRs from the https://mirdb.org/ database [3,4] to predict the target transcripts for hsa-miR0149-3p, hsa-miR-302a-5p, and hsa-miR-935. Since miRs can regulate gene expression by pairing them with particular mRNAs, usually in their 3′-termini, the https://mirdb.org/ database [3,4] was then used to predict the target transcripts of hsa-miR0149-3p, hsa-miR-302a-5p, and hsa-miR-935 that are upregulated in EVs from TB cells and are enriched in the brain and nervous tissues.

Supplementary File S5. ClueGO [5], which is a Cytoscape plug-in, was used to determine the gene ontology and pathways that are predicted to be affected by mRNAs and targeted by differentially expressed miRs in TB EVs vs. iPSC EVs.

Supplementary File S6. All the differentially expressed genes in NPCs treated with EVs from TB or iPSC or not treated with any EVs (control NPC). Based on a fold change ≥ 1.5 and q value (FDR) ≤ 0.05, there were 115 differentially regulated genes common to treatments with either kind of EVs, including 102 uniquely associated with treatment with EVs from iPSC and 38 associated with EVs from TB cells.

Supplementary File S7. To understand the interactions between miRs and lncRNA changes in TB-derived EVs and transcriptomic changes in NPCs treated with these structures, the NcPath [10] database, which provides KEGG [11] pathway associations, was used. This approach established

linkages between 26 miRs, 185 genes, and 786 lncRNAs. These are listed in the tab labeled "Biotype Counts". The last tab, labeled "DEG's Pathway Enrichment", lists the KEGG pathways affected by these interactions.

Supplementary Video S1. A 3D confocal video of human NPCs with internalized human TB EVs 40 minutes post-treatment. TB EVs stained in red. The nuclei of NPCs stained with DAPI and NPC cell processes stained with phalloidin.

Supplementary Video S2. A 3D confocal video of human NPCs with internalized human iPSC EVs 40 minutes post-treatment. TB EVs stained in red. The nuclei of NPCs stained with DAPI and NPC cell processes stained with phalloidin.

References Cited in this Section:

1. Jain, A.; Tuteja, G. TissueEnrich: Tissue-specific gene enrichment analysis. *Bioinformatics* **2019**, *35*, 1966-1967, doi:10.1093/bioinformatics/bty890.
2. Patil, A.H.; Halushka, M.K. miRge3.0: a comprehensive microRNA and tRF sequencing analysis pipeline. *NAR Genomics and Bioinformatics* **2021**, *3*, lqab068, doi:10.1093/nargab/lqab068.
3. Chen, Y.; Wang, X. miRDB: an online database for prediction of functional microRNA targets. *Nucleic Acids Res* **2020**, *48*, D127-D131, doi:10.1093/nar/gkz757.
4. Liu, W.; Wang, X. Prediction of functional microRNA targets by integrative modeling of microRNA binding and target expression data. *Genome Biol* **2019**, *20*, 18, doi:10.1186/s13059-019-1629-z.
5. Bindea, G.; Mlecnik, B.; Hackl, H.; Charoentong, P.; Tosolini, M.; Kirilovsky, A.; Fridman, W.H.; Pagès, F.; Trajanoski, Z.; Galon, J. ClueGO: a Cytoscape plug-in to decipher functionally grouped gene ontology and pathway annotation networks. *Bioinformatics* **2009**, *25*, 1091-1093, doi:10.1093/bioinformatics/btp101.

Disclaimer/Publisher's Note: The statements, opinions and data contained in all publications are solely those of the individual author(s) and contributor(s) and not of MDPI and/or the editor(s). MDPI and/or the editor(s) disclaim responsibility for any injury to people or property resulting from any ideas, methods, instructions or products referred to in the content.
